# P-1830. The First-in-Class Anti-Staphylococcal Antibiotic Afabicin Desphosphono is Not Associated With *Clostridioides difficile* Infection in an in vitro Human Gut Model

**DOI:** 10.1093/ofid/ofae631.1993

**Published:** 2025-01-29

**Authors:** Charmaine Normington, Emma Clark, Karen Bentley, Kimrun Kaur, Joy Bromley, Chiedza Chogugudza, William Spittle, Duncan Ewin, Ricardo Chaves, David Cameron, Mark H Wilcox, Caroline Chilton

**Affiliations:** 1) University of Leeds, Leeds, England, United Kingdom; Leeds Teaching Hospitals Trust, Leeds, England, United Kingdom; University of Leeds, Leeds, England, United Kingdom; University of Leeds, Leeds, England, United Kingdom; University of Leeds, Leeds, England, United Kingdom; University of Leeds, Leeds, England, United Kingdom; University of Leeds, Leeds, England, United Kingdom; University of Leeds, Leeds, England, United Kingdom; Debiopharm International, Lausanne, Vaud, Switzerland; Debiopharm International, Lausanne, Vaud, Switzerland; LEEDS TEACHING HOSPITALS & UNIVERSITY OF LEEDS, LEEDS, England, United Kingdom; University of Leeds, Leeds, England, United Kingdom

## Abstract

**Background:**

Antibiotic use is commonly associated with disruption of the healthy human gut microbiota (termed dysbiosis). Dysbiosis can diminish colonisation resistance and increase susceptibility to *Clostridioides difficile* infection (CDI). Use of the pathogen-specific anti-staphylococcal afabicin was not associated with dysbiosis in animals and healthy volunteers (Nowakowska et al, 2023). Here, we assessed whether this microbiota-sparing property of afabicin would limit CDI in a clinically reflective *in vitro* human gut model.

**Methods:**

A well-established gut model was used as described previously (Baines et al, 2005). Four independent models were seeded with human fecal slurry (d0) and inoculated at d14 and d21 with *C. difficile* spores (10^7^ CFU/mL). Two-week treatments were assessed beginning on d21 including (i) a CDI-positive control antibiotic levofloxacin (149.55 mg/L once daily), (ii) afabicin desphosphono high dose (35.7 mg/L twice daily [BID]), (iii) afabicin desphosphono mid dose (17.9 mg/L BID) and (iv) a vehicle control (1% DMSO BID). Endpoints for the model included facultative and obligate anaerobe counts, total viable and spore *C. difficile* counts and *C. difficile* toxin titre and were monitored daily until d63.

**Results:**

Each of the afabicin desphosphono treated models displayed colonisation resistance and no evidence of *C. difficile* germination or toxin production for the duration of the study. Further, no relevant changes in major facultative and obligate anaerobe groups were observed, indicating limited dysbiosis, similar to the vehicle control. In contrast, following a pre-treatment period where colonisation resistance was demonstrated (i.e. no germination following *C. difficile* inoculation), levofloxacin treatment resulted in dysbiosis characterized most notably by reductions (∼5log_10_ CFU/mL) in facultative anaerobes including lactose fermenting Enterobacteriaceae. Dysbiosis caused by levofloxacin led to CDI including *C. difficile* germination and toxin production.

**Conclusion:**

Findings from this study provide further support for the microbiota-sparing property of afabicin and suggest that limited off-target microbiota effects associated with afabicin treatment may reduce incidence of CDI when used clinically.

**Disclosures:**

**Charmaine Normington, PhD**, Debiopharm International S.A.: Grant/Research Support **Ricardo Chaves, n/a**, Debiopharm International S.A.: Salary **David Cameron, PhD**, Debiopharm International S.A.: Salary **Mark H. Wilcox, MD**, Astra Zeneca: Advisor/Consultant|Debiopharm International S.A.: Advisor/Consultant|Debiopharm International S.A.: Grant/Research Support|Ferring: Advisor/Consultant|GSK: Advisor/Consultant|GSK: Honoraria|Nestle: Advisor/Consultant|Paion: Advisor/Consultant|Pfizer: Advisor/Consultant|Pfizer: Grant/Research Support|Pfizer: Honoraria|Phico therapeutics: Advisor/Consultant|QPex Biopharma: Advisor/Consultant|Seres: Advisor/Consultant|Seres: Grant/Research Support|Seres: Lecture Fees|Summit: Advisor/Consultant|Summit: Grant/Research Support|The European Tissue Symposium: Advisor/Consultant|The European Tissue Symposium: Grant/Research Support|Tillotts: Advisor/Consultant|Tillotts: Grant/Research Support|Tillotts: Lecture Fees|Vedanta: Advisor/Consultant **Caroline Chilton, PhD**, Debiopharm International S.A.: Grant/Research Support

